# Gorilla Mothers Also Matter! New Insights on Social Transmission in Gorillas (*Gorilla gorilla gorilla*) in Captivity

**DOI:** 10.1371/journal.pone.0079600

**Published:** 2013-11-27

**Authors:** Eva Maria Luef, Simone Pika

**Affiliations:** 1 Max Planck Institute for Ornithology, Humboldt Research Group “Comparative Gestural Signalling,” Seewiesen, Germany; Centre for Coevolution of Biology & Culture, University of Durham, United Kingdom

## Abstract

The present paper describes two distinct behaviors relating to food processing and communication that were observed in a community of five separately housed groups of lowland gorillas (*Gorilla gorilla gorilla*) in captivity during two study periods one decade apart: (1) a food processing technique to separate wheat from chaff, the so-called puff-blowing technique; and (2) a male display used to attract the attention of visitors, the so-called throw-kiss-display. We investigated (a) whether the behaviors were transmitted within the respective groups; and if yes, (b) their possible mode of transmission. Our results showed that only the food processing technique spread from three to twenty-one individuals during the ten-year period, whereas the communicative display died out completely. The main transmission mode of the puff-blowing technique was the mother-offspring dyad: offspring of puff-blowing mothers showed the behavior, while the offspring of non- puff-blowing mothers did not. These results strongly support the role mothers play in the acquisition of novel skills and vertical social transmission. Furthermore, they suggest that behaviors, which provide a direct benefit to individuals, have a high chance of social transmission while the loss of benefits can result in the extinction of behaviors.

## Introduction

Learning via others is a fundamental building block of what is generally perceived as ‘culture’. When we define culture as patterns of behavior that are transmitted through social learning to become characteristic for a population [1], [2], we are presented with the challenges of how exactly ‘learning from others’ is achieved. Social transmission includes many behavioral facets that facilitate or enable the acquisition of skills through influences from the immediate social environment [3]. It is contrasted with the acquisition of behavior through individual learning and/or genetic determination [4]. While behavior of great apes may preclude an exclusive categorization of the underlying mechanisms, be it genetic, individual or social, there are a variety of indicators that can be interpreted to provide evidence for one hypothesis or another. The processes of learning are rarely directly observable but through a comparison of behavioral variations between and within populations, conclusions can be drawn as to how these behaviors may have spread [5], [6]. However, this so-called ‘method of exclusion’ [7], [8] has been criticized by some researchers arguing that insufficient attention has been paid to the difficulty of determining whether (i) ecological explanations can ever be definitively excluded as a source of behavioral variation and (ii) genetic differences are responsible for behavioral variation between groups and populations [9]–[11].

Social transmission can provide immense benefits to a group. Because contrary to individually acquiring every single physical or cognitive skill, transmitting information socially from one individual to another represents a much more efficient strategy to exploit ecological opportunities and to counter changing socio-ecological conditions [12]. To date, the debate over the role of social learning in the acquisition of behaviors of great apes, such as foraging skills and communication, is on-going [13]. In addition, implications that arise from social transmission and the discussion of ‘ape cultures’ are met with skepticism [14], [15]. In this paper, we aim to add to the discourse on social learning in apes by presenting findings that may shed new light on (a) why only certain behaviors in gorillas seem to be socially transmitted; and (b) the role mothers play for social transmission.

It has long been recognized that different forms of social learning exist which differ in the cognitive requirements that underlie their performance [16], [17]. Forms of social learning include (i) social facilitation where behavior of surrounding individuals activates matching behavior in an observing animal [3], and (ii) stimulus enhancement where the observing animal is led by the demonstrator to be increasingly exposed to a specific stimulus that causes his behavior to change accordingly [18]. Other forms of social learning are (iii) emulation where the observing individuals produce actions to achieve a similar effect to a demonstrator with objects in the environment, [18] and (iv) true imitation which requires to provide an exact motor copy of the behavioral sequences that are used by the demonstrator to achieve it [14]. So far emulation has been described as the dominant form of social learning in the tool acquisition of chimpanzees, with individuals paying less attention to the exact motor actions of the demonstrators than the general functional relation of the task [19]. In contrast, human children predominantly imitate exact action sequences of demonstrators rather than only those sequences that are necessary to achieve a certain goal [20]. The reason for this apparent difference between chimpanzees and children may lie with the fact that emulation and imitation are based on different underlying motivations in humans and nonhumans [21].

So far the majority of research has shown that social learning plays a role in the acquisition of physical cognitive skills such as the location and processing of food and antipredator behavior [22]–[25]. Different species of mammals, birds, fish, and insects are able to learn socially, such as for instance rats [26], bats [27], crows [28], guppies [29], and ants [30]. Concerning our closest living relatives, the nonhuman primates, the historically earliest examples stem from Japanese macaques (*Macaca fuscata*). Individuals were observed to use practices such as separating grains from sand by throwing a grain-sand mixture into seawater to collect the floating grains (so-called ‘wheat placer-mining’), and washing potatoes [31]. Unequivocal agreement, however, suggesting that these behaviors represent examples of social learning does not exist. Tomasello, for example, argued that potato-washing is learned individually even in the absence of social stimuli [32]. The recent discovery of food washing and placer mining in several groups of apes in captivity with no prior experience with the behaviors and no visual access to conspecifics engaging in the behavior, indicated that individual learning may indeed play a crucial role [33]. Concerning great apes, the majority of research has been focusing disproportionally on the common chimpanzee (*Pan troglodytes*), with examples ranging from behaviors such as nut-cracking and ant-dipping, to tool use for foraging and leaf-swallowing for self-medication (for a detailed overview see [7], [8]). Aside from food processing techniques, communicative gestures have also been implicated in the debate over social learning mechanisms. Behaviors such as the *grooming hand clasp* [34] or the *social scratch* [35] are likely candidates for transmission via copying from conspecifics [36]. Similarly, a variety of gestures of bonobos (*Pan paniscus*) have been investigated with sufficient evidence to support the hypothesis that social learning plays a role in their transmission, for example *chest beating, pirouetting* and *clapping* [37]–[39]. Thus, communicative behavior of bonobos seems to be a very promising domain for future research into social transmission. Furthermore, studies on orangutans and gorillas have also provided evidence for the social learning paradigm. Captive orangutans (*Pongo abelii, Pongo pygmaeus*), for instance, can learn the precise manner of extracting food through observing conspecifics [40], and diet choice of wild immature orangutans (*Pongo pygmaeus wurmbii*) has been shown to be largely based on observational learning from their mothers [41]. Concerning gorillas, investigations of social learning have been restricted to food processing techniques. Stoinski and colleagues [42] for instance showed that captive lowland gorillas (*Gorilla gorilla gorilla*) emulate human models when presented with food extraction problems. In addition, Watts [43] provided evidence that mountain gorilla infants (*Gorilla gorilla beringei*) in the wild learn which foods to choose by observing their mothers. Interestingly, a distinct feeding technique gorillas are using to process stinging nettles has stirred a recent debate: Byrne and Stokes [44] for instance argued that mountain gorillas acquire different techniques via social learning processes since infants match the nettle-technique their mothers have been using. Investigations into nettle-feeding techniques in captive gorillas even showed group-specific patterns, which provided further evidence for social learning [45]. Tennie and colleagues [46] compared the nettle-feeding techniques of three groups of lowland gorillas in captivity to findings of Byrne [47] on wild mountain gorillas. Contrary to the two other studies, they found that all animals showed the same technique when handling the nettles with differences existing only on the level of single actions during the manipulation of the nettles. The authors suggested that nettle-processing techniques are based on a combination of genetic disposition and individual learning [46]. This view is supported by Masi [48] who sees variety in nettle processing techniques as a result of ecological constraints which differ considerably between captive and wild gorilla.

To shed further light on this intriguing debate, the aim of the present study was twofold: First, we investigated the distribution and usage of a distinct food processing technique, the so-called puff-blowing technique, which has been suggested as likely candidate for social learning [49]. Second, since social learning has also been suggested to play a role in the acquisition of some communicative behaviors [7], [8], we examined the transmission path of a distinct male display, the so-called throw-kiss-display (Pika, personal observation), used to attract the attention of visitors. Since displays play such an important role in gorillas' every day life for instance during courtship and male-male competition, we predicted that this display might be a likely candidate for social transmission. Concerning displays, Parnell and Buchanan-Smith [50] showed that gorilla silverbacks who visit the forest clearing Mbeli Bai in the Republic of Congo use distinct displays to gain attention and to outcompete possible rivals by splashing water. In addition, a group-specific display in Cross River gorillas (*Gorilla gorilla diehli*) has been described by Wittiger and Sunderland-Groves [51] where individuals throw grass at human intruders. Furthermore, display behaviors in gorillas seems to be ‘contagious’ as it tends to elicit similar behaviors in other group members. Contagion (or ‘social facilitation’) appears when the performance of a certain behavior causes conspecifics to engage in the same behavior [52]. Concerning gorilla displays, through contagious behavior the threat signal becomes amplified, thereby serving the purpose of defending the group [53].

We chose two distinct behaviors that were observed in a gorilla community in 2000, the puff-blowing technique and the throw-kiss-display. The puff-blowing technique was used by three adult female gorillas [49], while the throw-kiss-display was performed by a single human-raised silverback only (Pika, personal observation). The present study compares the occurrences of puff-blowing and throw-kiss-displays during the two study periods one decade apart and investigates (a) whether the behaviors were transmitted within the respective groups; and if yes, (b) their possible mode of transmission. The exact mechanisms of how these behaviors have been learned cannot be elicited from our data, with the central question of our research being more generally whether some behaviors are more likely to be transmitted than others.

## Methods

### Data collection and coding

Feeding and communicative behavior of Western lowland gorillas from five captive groups at Howletts Wild Animal Park in Kent, United Kingdom, was observed during two study periods in 2000 and 2010. SP observed four mixed family groups during a period of eight weeks from June to August 2000 for approximately 4–6 hours per day. EML revisited three of the old groups and one new group from March to June 2010. All groups were observed for approximately 4–6 hours a day during morning and afternoon sessions, with a special focus on the use of the puff-blowing technique and the throw-kiss-display (Sampling rule: Behavior sampling; recording rule: continuous recording [54]). Video-clips were made from the visitors' areas with a Panasonic digital camcorder; the videos were edited on a MacBookPro using iMovie (Version 9.0.4) and Adobe Premiere Pro (Version 4.2.1). We used Microsoft-Excel (Version 14.3.8) to code the data according to age, sex, social group, family relations of the individuals, occurrences and subroutines of the behaviors.

### Ethics statement

The gorillas lived in indoor holding facilities and outdoor naturalistic exhibits between which they could alternate at any time throughout days and nights. The inside and outdoor areas contained climbing structures such as ropes and hammocks, manipulable items such as balls, and nesting and foraging material such as straw. The groups were regularly administered commercial primate food, fresh fruit, vegetables, and grains in a scatter feed. Water was available ad libitum in the enclosures. In addition, each morning the gorillas were individually given tea by the keepers. On occasion, honey pots were filled with honey or jam.

Our study was of a purely non-invasive nature with video recordings being taken from the visitors' areas near the enclosures, thereby aiming to not influence the daily behavioral regimen of the gorilla groups in any way. During all stages of the data collection, we took steps to ensure that the welfare of all animals was not compromised and no individual showed distress during any part of this study. The Aspinall Foundation, manager of Howletts and Port Lympne Wild Animal Parks, granted us permission to observe and film their gorilla groups in 2000 (to SP) and in 2010 (to EML). Due to the purely observational nature of our study, no ethic approval was necessary. The research adhered to the legal requirements of the country in which it was conducted and followed the recommendations of the ‘Animals (Scientific Procedures) Act 1986’ as published by the government of the United Kingdom and the principles of “Ethical Treatment of Non-Human Primates”, as stated by the American Society of Primatologists.

### Subjects

The animals and groups observed during the two study periods are listed below (see Tables 1 and 2). In 2000, each group was observed for 35 hours on average. In 2010, each group was observed for 20 hours on average. Tables 1 and 2 provide detailed information about the observed animals during both study periods in 2000 and 2010 as well as their sex, their age, and whether or not they performed the two behaviors.

**Table 1:** Observed animals during the study period 2000.

Group 1 (2000)	Group 2 (2000)
KOUILLOU, M, 17	Kifu, M, 13
**Tamba, F, 14**	Sidonie, F, 28
*Kwimba, F, 2*	Tebe, F, 20
Matibe, F, 12	*Kebu, F, 5 mo*
Jubi, F, 10	**Sounda, F, 16**
Mambi, F, 10	*Sanki, F, 2*
Emba, F, 9	**Sangha, F, 15**
	*Kanghu, M, 1*
	Bamilla, F, 13
	Tambabi, F, 14
	*Kifta, F, 4 mo*

Group 3 (2000)	Group 4 (2000)
Kijo, M, 25	Bitam, M, 29
Mushie, F, 31	Mouila, F, 40
*Komu, M, 3*	*Tamiela, F, 3*
Founa, F, 29	Baby Doll, F, 39
Shumba, F, 20	*Tamidol, F, 2*
*Kumbo, M, 5*	Juju, F, 38
*Kimba2, F, 2*	*Bubu, M, 3*
Juma, F, 19	Kabinda, F, 18
*Kidjoum, M, 3*	*Bandi, F, 3*
Dihi, F, 16	
*Kihi, M, 5*	
*Kidiki, F, 1*	

Observed animals [sex (F = female, M = male); age in years or months (mo)] during the study period in 2000. Animals showing the throw-kiss-display are marked in capital letters; animals showing puff-blowing are marked in bold. Offspring are indicated in italics and underlined below their mothers. Visual access was not given between the enclosures.

**Table 2:** Observed animals during the study period in 2010.

Group 1 (2010)	Group 2 (2010)
Kouillou, M, 27	Kifu, M, 23
**Tamba, F, 24**	Sidonie, F, 38
***Baloo, M, 6***	Tebe, F, 30
*Otana, M, 9*	*Ebeki, M, 7*
Matibe, F, 22	**Sangha, F, 26**
**Jubi, F, 20**	**Bamilla, F, 23**
**Mambi, F, 20**	Tambabi, F, 24
***Imbi, F, 10***	*Kifta, F, 10*
***Boula, F, 3***	**Sounda (†)**
*MahMah, F, 8*	***Oundi, F, 4***
*Mumbou, M, 1*	
Emba, F, 19	
*Imbizo, M, 5*	

Group 3 (2010)	Group 5 (2010)
Mushie, F, 41	Djanghou, M, 17
*Bitono, M, 9*	**Boma, F, 14**
*Popa, M, 6*	*Nkoumou, M, 1*
Shumba, F, 30	Kidiki, F, 11
*LouLou, F, 6*	**Kimba2, F, 12**
*Joshie, M, 3*	***Masindi, F, 4***
**Juma, F, 29**	*Affy, F, 1*
Dihi, F, 26	**Kwimba, F, 13**
*Jah, M, 8*	Tamidol, F, 12
*Kiju, M, 1*	(Sanki, F, 12)
	***Kisane, M, 4***

Observed animals [sex (F = female, M = male); age in years or months (mo)] during the study period in 2010. Animals showing the throw-kiss-display are marked in capital letters; animals showing puff-blowing are marked in bold; the animal in parentheses was not observed in this study. Female Sounda is indicated to have died before the second observation period (†). Offspring are indicated underlined and in italics below their mothers. Groups 1 to 3 are the same groups as in 2000; group 5 is a new family group. Visual access was not given between the enclosures.

In 2000, we observed four family groups (groups 1 to 4). The three originally described puff-blowers, Tamba, Sounda and Sangha, had lived in groups 1 and 2. Tamba was born at Howletts, while Sounda (died in 2007) and Sangha were wild-caught. They had been brought to Howletts at the age of two years and had always lived together in the same group. It is possible that Tamba had once lived with Sounda and Sangha in the same group or in an adjacent enclosure with visual access. When our second study period started in 2010, the silverbacks Kijo (died in 2006) and Bitam (died in 2008) had died. Kijo's group (group 3) was living without a silverback, while Bitam's group (group 4) had been dissolved. In 2010, we observed a new family group (group 5), consisting of several adult female offspring from other Howletts groups.

### Described behaviors

#### Puff-Blowing

The puff-blowing technique consists of puffing/blowing air with the mouth onto a mixture of oat grains and chaff in order to separate out the oat grains [49]. Video S1 shows puff-blowing sequences of an adult female.

#### Throw-Kiss-Display

The throw-kiss-display is characterized by bringing one hand to the mouth before quickly flinging the hand away while, at the same time, producing a smacking sound (see video S2). The routine is similar to humans blowing kisses and was previously described by Tanner [55] in a human-raised female gorilla. During the throw-kiss-display at Howletts many characteristics of the typical male-gorilla display are shown, including the so called stiff stance, turning the head away thereby only glancing at the recipient and running in a distinct direction [53], [56].

### Statistical analyses

#### Reliability

Twenty percent of the video data was checked for accuracy by a second observer. The reliability test was conducted using Cohen's Kappa, the coefficient of which is defined on a square r×r contingency table, measuring the agreement of two independent observers and correcting for the possibility of chance agreement. The two raters agreed with a kappa value of 0.82, which is considered an “excellent agreement” [57].

#### Parametric statistics

To analyze whether the probability of an individual to perform the puff-blowing technique was influenced by whether its mother showed this behavior or the number of group mates displaying this behavior, we used a Generalized Linear Mixed Model (GLMM [58]) with binomial error structure and logit link function. Into this we included the mothers' behavior (puff-blowing yes/no), age of subjects, and the number of adults per group who perform the technique as fixed effects. We predicted that a higher number of adult puff-blowers per group would lead to a higher rate of puff-blowing youngsters per group if horizontal transmission played a role. Since the effects of mother behavior or the number of adult puff-blowers in the group could show up only after a certain age, we included also the interactions with age into the model. Data on the visual access between the enclosures was not included since the animals could not see each other during feeding sessions on the ground and horizontal transmission between groups could therefore be excluded. The model was calculated with data of all offspring that were still living in their native groups with their mothers and had never been transferred to another group. Mother identity and social group were included as random effects. As an overall test of the effects of mother behavior and the number of adult puff-blowers in the group (either in the form of interactions with age or as main effects), we initially compared the full model as described above with a null model comprising only age and the random effect of mother [59] using a likelihood ratio test [60].

Prior to running the model, we square-root-transformed age to achieve a more symmetric distribution and then z-transformed age and the number of adult puff-blowers in the group to a mean of 0 and a standard deviation of 1. The model was implemented in R [61] using the function lmer of the R-package lme4 [62]. Likelihood ratio tests were conducted using the function *anova* with the argument test set to *Chisq*. The sample size for this analysis was 23 subjects of 13 mothers. Since only offspring of mothers using the puff-blowing technique also showed the behavior, the model had some complete separation issues [63]. We hence determined P-values for the individual effects using likelihood ratio tests.

## Results

In 2000, we observed the puff-blowing technique in three adult individuals [49] and none of the offspring. Since the technique is not performed reliably before the age of three, several gorilla infants during that study period were too young to use it. The throw-kiss-display was recorded in a single silverback.

Due to the foci of our studies being on gestural communication [64], [65] and thus distinct individuals, we were not able to systematically investigate puff-blowing across all individuals and groups. It is therefore possible that we may have missed the performance of skilled animals, who used the technique relatively infrequent during feeding and/or searching for grain. Nevertheless, with our focal samples we were able to capture an accurate representation of the animals' behaviors, including their food processing techniques.

In 2010, we observed puff-blowing in 15 individuals but did not record a single instance of the throw-kiss-display. By the start of our second study period, 13 additional individuals had acquired the puff-blowing technique and were performing it on a regular basis (two of the original three puff-blowers from 2000 were still alive and were still using the technique). While the throw-kiss-display was a common element of silverback Kouillou's display in 2000, it was never observed in any other animal during that observation period. In 2010, neither Kouillou nor any other individual were observed to perform the throw-kiss-display.

**Figure 1:**
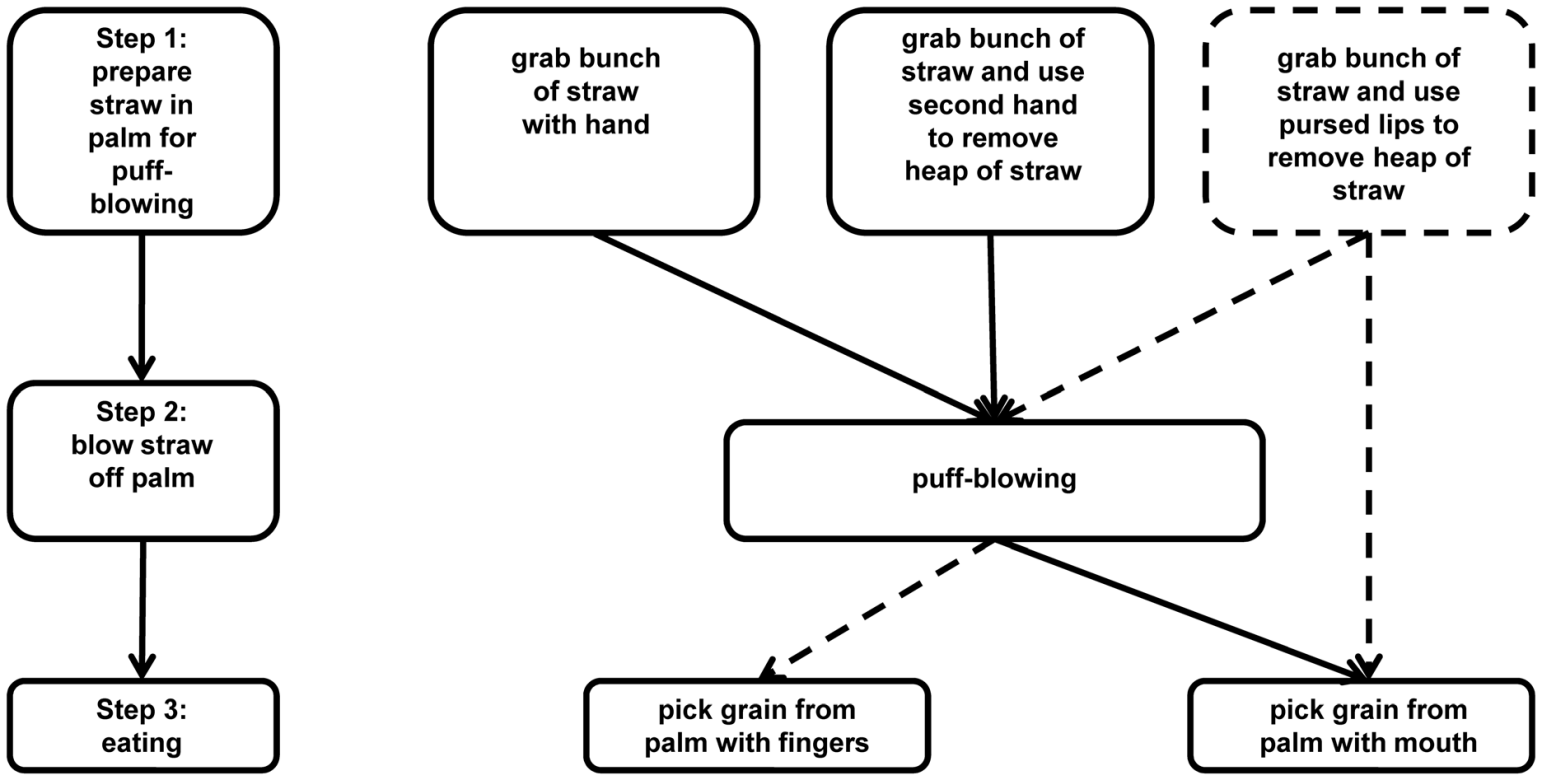
Details of the puff-blowing technique. Shows the puff-blowing technique in detail, which consists of several subroutines.

When analyzing the puff-blowing technique in detail, we found that it consists of several subroutines (see Figure 1). Except for two idiosyncratic variants, all animals performed puff-blowing according to the same sequential pattern and with relatively similar manual elaboration. puff-blowing spread from three to 15 individuals during one decade and the behavior appeared cumulatively in mothers and their offspring. In the observed groups in 2000 and 2010, only offspring of puff-blowing mothers used the technique, whereas none of the offspring of non- puff-blowing mothers did. There were three offspring who, despite having puff-blowing mothers, were never observed to perform the technique. Concerning the adult puff-blowers (i.e. mothers) in the groups, we do not have information concerning their learning of the technique.

### Statistical results

To investigate whether the mothers' performance had a significant influence on the offspring's use of the technique, we carried out a GLMM [58]. Overall the full model was highly significant as compared to the null model (χ^2^ = 11.63, df = 4, P = 0.020). After removal of the non-significant interactions between mothers' behavior and age (χ^2^ = 0.20, df = 1, P = 0.658) and the number of adult group mates using the puff-blowing technique and age (χ^2^ = 0, df = 1, P = 1), we found that the probability of puff-blowing was larger for subjects that had a mother showing the behavior than for subjects having a mother not showing the behavior (χ^2^ = 5.68, df = 1, P = 0.017). In addition, the number of puff-blowing adults per group had no significant effect on the spread of the technique (χ^2^ = 0.059, df = 1, P = 0.808).

### Descriptive results

Our analysis of the throw-kiss-display remains descriptive. Silverback Kouillou was recorded to perform the display 15 times between June and August 2000. By 2010, he did not use the behavior and it was also not performed by any other individual in Kouillou's group or in any of the other observed groups at Howletts.

## Discussion

The aim of the present paper was to add to the current debate on social learning in nonhuman primates by investigating whether (1) some behaviors are more likely to be transmitted than others, and if yes, (2) their possible mode of transmission. To do so, we focused on two distinct behaviors, one food processing technique (puff-blowing) and one communicative behavior (throw-kiss-display). Both behaviors had been observed on a regular basis in a gorilla community in 2000. We re-visited the same community ten years later to investigate the possible transmission paths of these two behaviors. Only the puff-blowing technique was still used by individuals of the gorilla community, while contrarily the throw-kiss-display had completely died out with even the originator not showing this behavior anymore. The use of puff-blowing increased from three to fifteen individuals during the ten years between the two study periods. These individuals lived in five groups, with nine puff-blowing mothers, six puff-blowing offspring, two non-puff-blowing individuals of skilled mothers and not a single instance of a puff-blowing offspring of a non-puff-blowing mother.

Four hypotheses may account for the spread of the puff-blowing technique. First, genetic differences may be responsible for certain individuals using the technique, while others did not. If this hypothesis were true, then we would expect to observe a non-random usage of the puff-blowing technique with only individuals of the same origin using this technique. This hypothesis however could not be verified, since the gorillas at Howletts originate from a variety of different places (Cameroun, Central African Republic, Republic of Congo, and various zoos, such as Jersey Zoo or Zoo Apenheul) with puff-blowers and non-puff-blowers randomly distributed across genetic lineages.

Second, differences in ecological conditions may explain the spread of the puff-blowing technique. This hypothesis suggests that considerate differences concerning housing and feeding conditions existed between the study groups, resulting in a positive correlation between straw and grain distribution and amount of puff-blowers per group. This prediction does not accord with our findings: Although not identical, housing conditions at Howletts are very similar between the gorilla groups, with the facilities' architectural designs being nearly identical in size and fitting of the enclosures, consisting of equipment such as climbing structures and enrichment opportunities (see also method section). All gorilla groups undergo the same feeding regimen every day and, most importantly, the availability of straw and grains is comparable for all individuals. In addition, every group consisted of an average of 3.5 puff-blowers and the use of the technique was not restricted to single groups only. Thus ecological constraints cannot satisfactorily explain why some gorillas used the puff-blowing technique while others did not. Non-puff blowers either did not separate grains from straw or picked up the grains with their fingers when they were on ground without straw.

Third, each puff-blowing individual may have invented the technique independently. In a shared environment with similar opportunities for every individual, different gorillas may invent the same behavior [66]. If this hypothesis were true, we would have predicted to find an unbiased distribution of puff-blowers in the groups. However, this was not the case, because our data showed cumulative occurrences of the behavior within closely related animals.

Fourth, the transmission of the puff-blowing technique may be due to a social learning process, where individuals acquire behavior based on observational learning of others [22]. If this hypothesis were true, we predicted to find the probability of puff-blowing being larger in individuals with skilled group members from which they could have learned the technique. This prediction corresponds with our results: puff-blowing was most frequently observed in mothers and their offspring. These finding strengthen the so called “family-model of social transmission” [67], which predicts that individuals learn behaviors from family members and that knowledge transfer occurs within family units. Gorilla infants spending the majority of their first years in close proximity to their mothers, are offered many opportunities for social learning of various behaviors [68]. By taking part in the mother's activities, infants of puff-blowing mothers come into contact with the feeding technique early, which may facilitate their acquisition of the technique. The family model is a common explanation for social learning of various behaviors in different species [69]–[71]. Particularly favorable conditions exist for social learning in kin as they spend more time in close proximity to one another than they do with non-related individuals, and thus share a more similar environment with one another. This factor significantly facilitates the learning of behaviors from close kin and can account for many instances of social learning [23]. In fact, kin-biased learning provides advantages because copying behavior is most useful when social model and observer experience the same environment and share a genetic make-up due to similarities in environment requiring similar strategies in dealing with environmental challenges [72], [73]. Since the relationship between mother and offspring is a genetic as well as a social one, genetic influences may play some role as well. Similarly to all primate species, the mother-infant dyad in gorillas is the closest-knit union and the amount of time that is spent in close proximity to each other offers many opportunities for social transmission of behavior [68]. Maternal support for offspring is advantageous in evolutionary terms as mothers can gain indirect fitness benefits by increased fitness on the part of their offspring [74]. By acquiring a new food processing technique, such as puff-blowing, individuals can expand their diet and access more food sources (or forage more efficiently), which may significantly increase their adaptability to environmental conditions and ultimately their survival. Nonetheless, not all offspring of puff-blowing mothers adopted the behavior. In addition, among siblings some did use the technique while others did not. This may be explained by different underlying motivations in individuals that determine whether or not they socially learn from their mothers.

However, it is important to note that our study inferred the social transmission of puff-blowing by analyzing the distribution of the technique within groups and relating it to the relationship between the puff-blowing individuals. We can thus only speculate about the exact ontogeny of the behavior and its origins. It is possible that puff-blowing may have started with individual learning on the part of one or more animals and then spread through social learning to others in the groups. Concerning group compositions, information on past compositions, in particular before 2000, is not available at present and we can thus neither support nor refute hypotheses on individual or social learning of the puff-blowing technique in the mothers. The three original puff-blowers may have been co-housed at some point or may have been able to observe each other through neighboring enclosures. Similarly, the proficient mothers observed in 2010 may have learned the technique through horizontal transmission, i.e. by observing non-related group members and acquiring the technique based on their behavior. Alternatively, each adult may have independently invented the technique. The fact that the puff-blowing technique was also observed in a gorilla group in a zoo in Germany [75], which comprised of wild-caught individuals and offspring of wild-caught parents, suggests that individuals can invent the behavior given the right environmental circumstances. This point is further strengthened by the two idiosyncratic variants of the puff-blowing subroutines observed in our study community: (1) one female used her second hand to pick the grains from her palm after she had blown off the straw; and (2) a young male frequently used his pursed lips to push the heap of straw off his palm instead of blowing onto it. Thus, the finding that gorillas develop idiosyncratic variants of the puff-blowing technique provides strong evidence for their ability to invent and individually learn advantageous feeding techniques.

Concerning the ontogenetic development of the puff-blowing technique, we observed three juveniles performing the puff-blowing technique in an adult-like manner; going through all the steps of the technique in the correct order as it was displayed by skilled adults. Before that age, no infant gorilla was observed to puff-blow. This is consistent with previous findings suggesting that juvenile gorillas are generally proficient at foraging tasks [76].

In sum, our data indicate that vertical transmission of behavior from mothers to offspring plays a crucial role in the social transmission of the puff-blowing technique and probably feeding techniques in general. While some individuals may have learned the behavior from puff-blowers other than their mothers, there is evidence that vertical transmission does commonly take place and can account for many instances of social learning in our study population. These results support previous findings of Byrne and Byrne [77], who found that mountain gorilla mothers have a crucial impact on the acquisition of distinct feeding techniques of their offspring.

Contrary to the puff-blowing technique, the throw-kiss-display did not spread within the population. Even though display behavior occurs in individuals of various ages and ranks in gorillas and is generally ‘contagious’ [53], we did not observe a single instance of the throw-kiss-display in 2010. Concerning the origins of the display, we assume that it is related to the “food-request kiss” that was commonly produced by the first gorillas at Howletts [78]. John Aspinall, the founder of Howletts and Port Lympe Wild Animals Parks, described a “loud kissing sound” that the gorillas used to attract attention and to request food from caregivers in the 1970's [78]. Kouillou was reared by the Aspinall family from an early age and may have invented the throw-kiss-display during such feeding encounters, by combining elements of the typical gorilla male display with the “food-request-kiss”. Since feeding encounters are generally characterized by a relatively high degree of competition between group members, Kouillou may have used this unique combination to advertise his presence to the keepers and to repel possible competitors by intimidation [53]. When our observations started in 2000, the original “food-request-kiss” had already been given up by the gorillas, probably due to changed feeding procedures from individual to standardized provisioning. In addition, the use of the throw-kiss-display resulted in attracting the attention of visitors only rather than in a significant increase of feeding success (Pika, personal observation). Loosing its original meaning, function and benefits, Kouillou dropped the throw-kiss-display from his communicative repertoire in the subsequent years. Furthermore, the throw-kiss-display had a smaller founder population than the puff-blowing technique to begin with. This also could have contributed to its disappearance as such ‘founder effects’ regarding the number of individuals from which a behavior starts to spread, are influential upon the survival of socially learned information [79].

In conclusion, we propose that research on social learning abilities should be expanded to also include possible costs and benefits that an animal may suffer/gain from acquiring certain behaviors. puff-blowing for instance allows gorillas to exploit food resources more efficiently and thus may result in direct fitness benefits [49]. This is in contrast to the throw-kiss-display, whose function as an attention-getter and ‘intimidator’ during food begging decreased dramatically. A particular behavior may be evaluated based on the incurred costs and benefits and if the benefits outweigh the costs, such as for instance increased food access, incentives for social learning are given. The low profit that an unsuccessful display signal offers, as opposed to the high profit that a new feeding technique provides, may explain why puff-blowing was readily acquired by other group members while the throw-kiss-display disappeared entirely (however see [80]).

As social learning consists of many different cognitive processes, there are undoubtedly several parallel processes at work that determine whether a distinct ability can spread within a group. Our study thus provides an important contribution to current debates on social learning by strengthening the role mothers have on the behavioral performance and skills of their offspring. These findings will hopefully inspire and direct future research to investigate the factors influencing the motivational aspects triggering social learning on behalf of both, observers and models.

## Supporting Information

Video S1 **The puff-blowing technique.** Shows puff-blowing sequences of an adult female.
(M4V)

Video S2 **The throw-kiss-display.** Shows the throw-kiss-display of a silverback.
(MOV)
